# Effects of self-acupressure on quality of life and abdominal pain severity among patients with inflammatory bowel disease: A randomized sham-controlled trial

**DOI:** 10.1016/j.jaim.2024.101080

**Published:** 2025-03-25

**Authors:** Nahid Rajai, Zahra Abbasi, Amir Hosein Pishgooie, Fatemeh Teymouri, Mohammad Imanipour

**Affiliations:** a Department of Critical Care Nursing, Faculty of Nursing, Aja University of Medical Sciences, Tehran, Iran; b Shahid Chamran Hospital, Faculty of Nursing, Aja University of Medical Sciences, Tehran, Iran; c Department of Intensive Care Nursing, Faculty of Nursing, Aja University of Medical Sciences, Tehran, Iran; d Ph.D. in Health in Disasters and Emergencies, Assistant Professor, School of Nursing, Aja University of Medical Sciences, Tehran, Iran; e Department of Medical-Surgical Nursing, Faculty of Nursing, Aja University of Medical Sciences, Tehran, Iran

**Keywords:** Acupressure, Inflammatory bowel diseases, Integrative medicine, Pain management, Quality of life

## Abstract

**Background:**

Acupressure is being used by patients to relieve functional gastrointestinal disorders.

**Objective:**

The impact of acupressure on quality of life (QoL) and pain has been shown in patients with both acute and chronic conditions. However, its effects on individuals with inflammatory bowel disease (IBD) remain uncertain. This trial aims to explore the potential effects of self-acupressure on QoL and abdominal pain severity in IBD patients.

**Methods:**

This randomized sham-controlled trial involved 56 outpatients with IBD referred to the emergency department, who were randomly assigned to either an acupressure group or a sham group. All patients received standard care along with daily follow-up calls. Those in the acupressure group applied pressure at four specific points, while the sham group engaged in superficial self-touch at corresponding points. Both interventions occurred three times daily for four weeks. The quality of life was assessed using the IBDQ-9 questionnaire and a visual analog scale (VAS) at both baseline and endpoint.

**Results:**

The mean IBDQ-9 score was significantly higher in the acupressure group compared to the sham group at the trial's end (P < 0.001). Likewise, the mean VAS score was notably lower in the acupressure group at the endpoint (P < 0.001). Additionally, the change in mean IBDQ-9 scores from baseline to endpoint favored the acupressure group significantly (P < 0.001), and the same significant difference was observed for the mean VAS score change (P < 0.001).

**Conclusion:**

Daily follow-up-guided self-acupressure may improve quality of life and alleviate abdominal pain in IBD sufferers.

## 1 Introduction

Inflammatory bowel disease (IBD) is a chronic condition characterized by recurrent inflammation of the gastrointestinal tract (GIT). It primarily includes Crohn's disease (CD) and ulcerative colitis (UC). CD involves transmural inflammation that can affect any part of the GIT, while UC is marked by superficial inflammation of the rectum, extending into adjacent mucosa persistently [1]. The primary causes of IBD include abnormal immune responses, genetic factors, and epigenetics [2]. Although IBD can develop at any age, it typically begins between 15 and 30 years and affects both genders equally [3]. The IBD is more common in developed countries and has been increasing in prevalence in developing nations, particularly in the Middle East and Asia [4]. Approximately 2.5 million people in Europe and 1 million in the United States are estimated to have IBD each year [5].

IBD impacts all aspects of patients' lives, including occupational, personal, and social spheres, resulting in a significant socioeconomic burden [6,7]. Individuals with IBD face symptoms such as abdominal and joint pain, bloody stools, chronic diarrhea, and undesired weight loss, which negatively affect their daily activities [8]. Abdominal pain, experienced by approximately 70% of patients, has the most pronounced impact on everyday life [9,10]. Additionally, IBD typically requires lifelong treatment, monitoring, and adaptation, leading to psychosocial distress and further disruption to daily life [11]. Consequently, perceived quality of life (QoL) among people with IBD is significantly lower than that of healthy individuals [12].

For years, IBD treatment aims to reduce inflammation, prevent complications, improve symptoms, and minimize surgery needs [13]. The chronic nature of IBD and its relapsing-remitting characteristics make improving QoL a crucial focus in IBD management [14]. IBD treatment and care plans should be tailored to enhance patient QoL [15]. Recent evidence supports that integrated patient-centered care enhances health outcomes and quality of life for individuals with IBD [16]. Greater focus is needed on developing patient-centered care programs to enhance the quality of life for IBD sufferers.

Patients with gastrointestinal diseases like IBD increasingly turn to various complementary and alternative therapies (CATs) at different stages of their illness, often due to symptom recurrence and limited effectiveness of conventional treatments [17,18]. Acupressure and acupuncture, the most popular complementary and alternative therapies, have long been used by patients to relieve functional gastrointestinal disorders [19]. These modalities were commonly reported as CATs used by patients with IBD [20,21].

In acupressure, acupoints are stimulated using fingers, palms, or devices to balance energy, promote health, and prevent illness [22]. The philosophy of acupressure suggests that this CAT can promote calmness and psychological well-being by regulating neuro-hormonal responses [23,24]. Acupoint stimulation can modulate the autonomic nervous system by reducing sympathetic activity and increasing parasympathetic activity, thereby alleviating psychological issues and decreasing the need for sedatives [25,26]. Acupoint stimulation can reduce systemic inflammation and enhance immune function [27]. It also enhances blood flow and stimulates alpha brain waves, promoting relaxation and mitigating psychological effects [17,28]. According to the gate control theory of pain, acupressure transmits pleasurable stimuli to the brain four times faster than painful ones, effectively closing the nerve gates and preventing pain signals from reaching the brain [29].

Recent trials have documented the efficacy and safety of acupressure in managing quality of life and pain indicators across various health conditions. [30,31]. Recent reviews suggest that acupressure may effectively improve health outcomes, but they call for more rigorous studies across diverse populations. [[32], [33], [34]]. Research on acupressure and acupuncture among individuals with IBD has rapidly increased in recent decades [35,36]. More research is needed on the safety and efficacy of methods like acupressure for the IBD population. Previous studies have reported the benefits of acupuncture for alleviating anxiety and pain in IBD patients, which can greatly enhance quality of life [37]. No study has examined the effectiveness of acupressure on pain and quality of life (QoL) for IBD sufferers. Therefore, this research evaluated the impact of self-acupressure on QoL as the primary outcome and abdominal pain severity as the secondary outcome for IBD patients.

## 2 Materials and methods

This was a single-center, randomized, parallel-arm, sham-controlled trial with a pre- and post-test design, approved by the Ethics Board of Aja University of Medical Sciences (Tehran, Iran) under code IR.AJAUMS.REC.1399.197 and registered in the Iranian Registry of Clinical Trials (approval No. IRCT20210306050603N1).

### 2.1 Participants and setting

Outpatients with IBD were invited to participate in this trial conducted at the emergency department of Shahid Chamran Hospital in Tehran, Iran. This hospital was chosen due to its daily admission of 200–600 patients, with approximately 10% experiencing gastrointestinal disorders and abdominal pain.

Patients were eligible if they met the following criteria: 1) a confirmed diagnosis of IBD in their medical record, 2) aged between 18 and 60, 3) able to communicate proficiently in Persian with adequate literacy, and 4) qualification for completing study interventions requires approval from an assistant emergency physician. The exclusion criteria included: 1) a history of chronic diseases (e.g., autoimmune conditions and cancers) or psychological issues (e.g., stress, anxiety), 2) any injuries, deformities, or numbness at or near the acupoints, 3) pregnancy, 4) previous participation in acupressure programs or conducting similar interventions, 5) addiction to smoking or drugs, 6) use of analgesics, tranquilizers, sedatives, or anti-anxiety medications within 12 h before admission, and 7) missing more than seven intervention days.

### 2.2 Sample size and randomization

Using G-Power software (version 3.0.10, Means: Difference between two independent means; two groups), we estimated a sample size of 26 patients per study arm with a 95% confidence interval (α = 0.05), 80% power (β = 0.20), an anticipated effect size of 0.79 (hypothesized difference: 5.50), and a 1:1 group ratio. To account for a 10% attrition rate, we selected 28 patients per arm.

Outpatients referred for routine care and medical visits were consecutively selected using a convenience sampling approach from May to July 2021. Of the 72 samples assessed for eligibility, 56 were randomly assigned to either the acupressure group (n = 28) or the sham group (n = 28) through coin tossing. A nursing assistant handled the sampling and randomization, keeping all related information confidential until the trial concluded.

### 2.3 Blinding

The trial was conducted in an open-label manner, making it impossible to blind patients due to the nature of acupressure. Study outcomes were assessed using self-completed tools. To reduce bias, a superficial touch was applied to sham points, matching the intervention frequency of the acupressure group. Additionally, the first and second assistants responsible for recording outcomes and providing routine care were unaware of group allocations.Only the nursing assistant, who handled randomization and sampling, knew the allocation codes during the trial.

### 2.4 Measurement

The study tools included a demographic-clinical questionnaire, the IBDQ-9 for quality of life assessment in IBD patients, a visual analog scale for pain evaluation, and a form for reporting adverse effects.Patients completed the IBDQ-9 and VAS with the assistance of a blinded helper before and after the trial. In contrast, an assistant administered the two other study tools. The demographic-clinical questionnaire was filled out by interviewing patients and reviewing their medical records before randomization.Additionally, the adverse effects form was completed during daily telephonic follow-ups.

The Persian version of the IBDQ-9 was developed to assess patients' quality of life (QoL) as the primary outcome. The IBDQ-9, a brief version of the original IBDQ by Casellas et al. (2004) [38], is a self-administered questionnaire consisting of nine items.It evaluates patient QoL across four domains: gastrointestinal, systemic, emotional, and social issues. Responses are rated on a scale from one to seven (worst to best condition), yielding a total score of 9–63, where higher scores indicate better QoL. The Persian IBDQ-9 has demonstrated adequate validity and reliability [39]. In this survey, the tool demonstrated a Cronbach's alpha coefficient of 0.89 based on data from 56 recruited patients.

The VAS was concurrently used with the IBDQ-9 to assess the severity of patients' abdominal pain as a secondary outcome. This tool consists of a calibrated line with ten points, ranging from 0, labeled "I don't feel pain," to 10, labeled "I feel severe pain." [40]. Patients were asked to indicate their abdominal pain level using a line position. The VAS is a valid, reliable, and easily understood tool for measuring pain intensity [41].

### 2.5 Data collection and intervention procedures

Two study groups received standard care as per the recruitment hospital's protocol, delivered by a blinded nursing assistant and monitored by a blinded emergency physician. The experimental group underwent acupressure at four acupoints, while the sham group received a superficial touch at sham points. Both interventions and standard care were administered from the first day of admission and continued for four weeks. On the first day of patients' admission to the emergency department, their baseline quality of life and pain severity were recorded by an uninformed assistant. Another assistant then carried out the random allocation. Following this, patients in both groups received an in-person instructional session in a private room from a qualified nursing assistant who had completed specialized acupressure training led by an experienced acupressurist.

An assistant, distinct from those documenting the study outcomes and providing routine care, delivered the instructions. All patients received a brief overview of IBD along with its pharmacological and non-pharmacological treatments. They were trained in self-care and the study interventions through lectures, videos, demonstrations, role-playing, and question and answer. Each patient practiced the intervention twice, with accuracy checks to address any understatements of issues. Additionally, patients were instructed to take only prescribed medications and to avoid any actions outside the study protocol. They were also advised against conducting interventions immediately before or after meals, baths, and exercise.

At the end of the instructional session, patients were encouraged to follow the study interventions and given a pamphlet summarizing the instructions for further review. They were asked to return to the hospital one day after completing the four-week intervention, where their quality of life and pain severity were reassessed. During the intervention period, patients could visit the emergency department for routine care if needed. The acupressure group was scheduled to visit the hospital on even days, while the sham group was asked to come on odd days to prevent communication between the groups.

The intervention sessions were conducted three times daily (every 8 h) for four consecutive weeks, totaling 84 sessions. The first session occurred on the patients' admission day, following the instructional session. In subsequent sessions, patients carried out the interventions at home using the same approach. Throughout the four-week period, an assistant reminded patients of their home sessions through daily phone calls, during which any potential adverse effects were documented. The assistant also monitored patients' compliance and quality of intervention administration through these follow-ups. Additionally, all patients were asked to record at least one weekly video of their home sessions and share it with the assistant via private social media messages. If any patients missed a session or performed it incorrectly, the issues were addressed, and they were encouraged to adhere to the instructions for future sessions.

Acupoints were chosen based on the acupressurist's recommendations: 1) stomach meridian (ST), 2) kidney meridian (KID), 3) spleen meridian (SP), and 4) large intestine 4 (Li 4) (Table 1). Pressure was applied to each point using fingertip technique for 10–15 s. In the acupressure group, pressure began with deep breathing and continued until the patient experienced warmth, heaviness, swelling, or numbness at the points. In the sham group, participants touched the sham points superficially for similar durations and timings.

**Table 1:** Details of acupoints in the acupressure group.

Acupoints		Locations	Pictures
Stomach Meridian (ST)	ST 23	On the upper abdomen, 2 cm above the center of the umbilicus, 2 cm lateral to the anterior midline	
	ST 24	On the upper abdomen, 1 cm above the center of the umbilicus, 2 cm lateral to the anterior midline	
	ST 25	On the middle of the abdomen, 2 cm lateral to the umbilicus	
	ST 26	On the lower abdomen, 1 cm below the center of the umbilicus, 2 cm lateral to the anterior midline	
	ST 27	On the lower abdomen, 2 cm below the center of the umbilicus, 2 cm lateral to the anterior midline	
	ST 28	On the lower abdomen, 3 cm below the center of the umbilicus, 2 cm lateral to the anterior midline	
Kidney Meridian (KID)	KID 2	On the medial part of the foot, below the tuberosity of the navicular bone, at the junction of the red and white skin	
	KID 3	On the medial part of the foot, posterior to the medial malleolus, in the depression between the tip of the medial malleolus and tendo-calcaneus	
	KID 4	On the medial part of the foot, posterior and inferior to the medial malleolus, in the depression anterior to the medial side of the attachment of tendo-calcaneus	
	KID 5	On the medial part of the foot, posterior and inferior to the medial malleolus, 1 cm directly below KID 3, in the depression of the medial side of the tuberosity of the calcaneum	
Spleen Meridian (SP)	SP 3	On the medial part of the foot, in the depression posterior and inferior to the proximal metatarsodigital joint of the big toe, at the junction of the red and white skin	
	SP 4	On the medial part of the foot, in the depression distal and inferior to the base of the first metatarsal bone	
	SP 5	On the depression distal and inferior to the medial malleolus, the midpoint between the tuberosity of the navicular bone and the tip of the medial malleolus	
	SP 6	On the medial part of the lower leg, 3 cm above the medial malleolus, on the posterior border of the medial aspect of the tibia	
Large intestine 4 (Li 4)		On the dorsum of the hand, between the first and second metacarpal bones, approximately in the midpoint of the second metacarpal bone on the radial side, in the belly of the first interosseus dorsalis muscle	

**Note:** Acupressure on KID, SP, and Li4 acupoints was applied on both sides.

### 2.6 Statistical methods

**Fig. 1:**
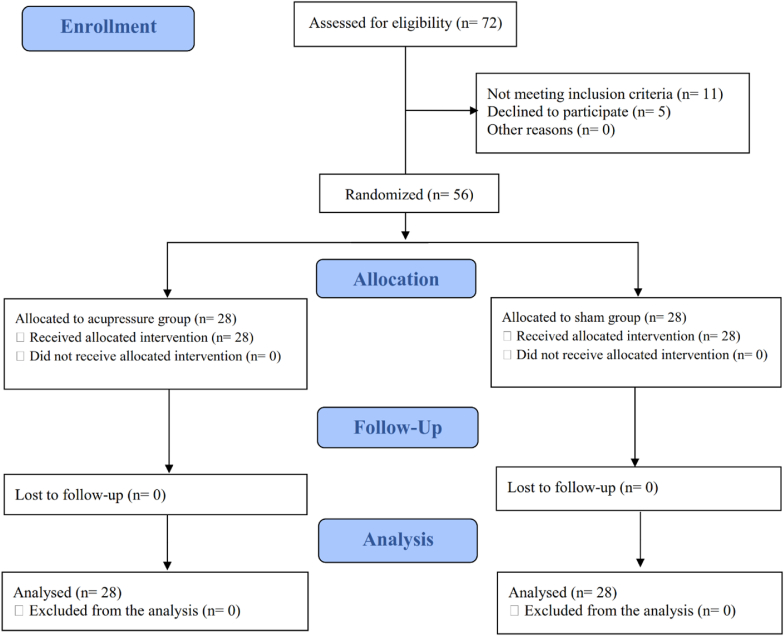
CONSORT flow diagram of the study participants.

We intended to address missing data through intention-to-treat analysis, using multiple imputations. However, all randomized patients completed the trial and were included in the final analysis (Fig. 1). Data analysis was conducted withthe Statistical Package for Social Sciences software (IBM SPSS Statistics, version 16.00, IBM Corp., New York, USA). We used independent and paired samples t-tests to compare study outcomes between and within groups. Additionally, we applied the independent samples *t*-test to assess mean changes in outcomes from baseline to endpoint. A P-value of <0.05 was considered statistically significant for all tests.

### 2.7 Ethical considerations

All patients were informed about the trial objectives and assured that their personal data would remain confidential and used solely for research. They were informed that they would receive either a superficial touch or acupressure without interruption of their routine care. Complete informed consent, both verbal and written, was obtained from all patients for participation and publication.

## 3 Results

### 3.1 Patient characteristics

In the acupressure group, 46.4% of patients were aged 25–45, compared to 53.6% in the sham group. No statistically significant differences in descriptive characteristics were found between the two groups (Table 2).

**Table 2:** Demographic and clinical characteristics of the study groups.

Qualitative variables		Acupressure group (*n* = 28)	Sham group (*n* = 28)	*Between-group P-value*
		N (%)	N (%)	
**Gender**	Male	13 (46.4)	14 (50.0)	1.00 a
	Female	15 (53.6)	14 (50.0)	
**Marital Status**	Single	15 (53.6)	12 (42.9)	0.614 b
	Married	10 (35.7)	14 (50.0)	
	Divorced	3 (10.7)	2 (7.1)	
**Job status**	Jobless	12 (42.9)	8 (28.6)	0.522 b
	Employed	16 (50.0)	18 (64.3)	
	Retired	2 (7.1)	2 (7.1)	
**Educational level**	Below diploma	1 (3.6)	2 (7.1)	0.852 b
	Diploma	7 (25.0)	10 (35.7)	
	Graduation and above	20 (71.4)	16 (57.2)	
**History of hospitalization**	Yes	22 (78.6)	23 (82.1)	1.00 a
	No	6 (21.4)	5 (17.9)	
**Diet type**	Regular	13 (64.4)	14 (50.0)	0.929 b
	Diabetic	2 (7.1)	1 (3.6)	
	Low salt-fat	3 (10.7)	2 (7.1)	
	Fast food	5 (17.9)	7 (25.0)	
	Without lactose/spices	5 (17.9)	4 (14.3)	
**Quantitative variables**		**Mean ± SD**	**Mean ± SD**	
**Age (years)**		29.68 ± 9.62	31.79 ± 11.50	0.461^c^
**Duration of IBD (years)**		4.14 ± 3.67	3.39 ± 2.00	0.349^c^

**Note:** Quantitative variables have been expressed as mean ± standard deviation (SD), while qualitative variables have been presented as number (percentage).

a Fisher's exact test.

b Chi-square test.

c Independent samples *t*-test.

### 3.2 Main outcomes

The mean scores for patients' quality of life (QoL) and pain severity in both study groups are shown in Table 3. The independent samples *t*-test revealed no significant differences in baseline mean scores for IBDQ-9 and VAS (P = 0.462, P = 0.321). However, the post-test mean score for IBDQ-9 in the acupressure group was significantly higher than in the sham group (P < 0.001). Likewise, the mean VAS score was significantly lower in the acupressure group at the endpoint (P < 0.001). The paired samples *t*-test identified a significant improvement in the acupressure group's post-test IBDQ-9 scores compared to baseline (P < 0.001), as well as in VAS scores (P < 0.001). In contrast, no significant intra-group differences were found in the sham group (P = 0.213 for IBDQ-9, P = 0.326 for VAS).

**Table 3:** Comparison of quality of life and abdominal pain severity in the study groups.

Variables		Acupressure group (*n* = 28)	Sham group (*n* = 28)	*Between-group results*	
				P-value^c^	Effect size^d^
**Quality of life**^a^	Before intervention	32.64 ± 7.66	30.9 ± 9.86	0.462	0.197
	After intervention	44.07 ± 5.51	31.82 ± 8.15	<0.001	1.760
	*Within-group P-value*^e^	<0.001	0.213		
**Pain severity**^b^	Before intervention	6.79 ± 1.77	7.29 ± 1.96	0.321	0.267
	After intervention	2.11 ± 1.85	7.14 ± 2.13	<0.001	2.521
	*Within-group P-value*^e^	<0.001	0.326		

**Note:** All values have been expressed as mean ± standard deviation (SD).

a Quality of life was measured by a quality of life questionnaire for patients with inflammatory bowel disease (IBDQ-9): the total score ranges from 9 to 63, with higher scores indicating better quality of life.

b Pain severity was recorded by a 0–10 visual pain rating scale.

c Independent samples *t*-test.

d Cohen's d.

e Paired samples *t*-test.

The independent samples *t*-test revealed a significant inter-group difference in the mean change of IBDQ-9 scores from baseline to post-test (11.42 ± 6.69 vs. 0.92 ± 3.84, P < 0.001). Similar results were observed for the mean difference in VAS scores (˗4.67 ± 1.18 vs. ˗0.14 ± 0.75, P < 0.001). The increases in IBDQ-9 scores and decreases in VAS scores were notably greater in the acupressure group compared to the sham group.

### 3.3 Adverse effects

No participants experienced adverse effects from either the acupressure or sham interventions.

## 4 Discussion

This study examined the effects of a 4-week self-acupressure intervention on quality of life (QoL) and abdominal pain severity in Iranians with inflammatory bowel disease (IBD). The results showed that patients receiving usual care plus acupressure reported a higher QoL than those receiving routine care plus a sham intervention (simple touch). Additionally, the acupressure group experienced a significantly greater improvement in QoL from baseline to endpoint. The study also found a notable reduction in pain severity in the acupressure group at the trial's conclusion compared to the sham group, with a significantly greater decrease in pain severity from baseline in the acupressure group. These findings suggest that acupressure could effectively enhance QoL and alleviate pain in IBD patients.In this study, the IBDQ-9 score, which assesses gastrointestinal, systemic, emotional, and social aspects, significantly improved at the endpoint for patients in the acupressure group compared to the sham group. These findings support existing evidence of acupressure's effectiveness in enhancing quality of life in patients with various gastrointestinal issues [42,43]. A Randomized Controlled Trial (RCT) showed that perineal self-acupressure combined with standard treatments significantly enhanced self-reported quality of life (QoL) in patients with functional constipation compared to standard treatments alone [42]. Additionally, another trial demonstrated that acupressure notably improved the QoL of patients with advanced gastroenteric tumors relative to supportive treatments [43].

The current study supports previous trials demonstrating the analgesic efficacy of acupressure for gastrointestinal conditions. In line with our findings, a recent RCT involving colon and pancreatic cancer patients undergoing chemotherapy showed significantly lower VAS scores in those who participated in a 4-week self-acupressure program (16 sessions on four pressure points) compared to those in a standard care program [44]. Recent RCTs have shown that post-operative acupressure significantly reduces abdominal pain VAS scores and gastrointestinal immotility after cesarean sections compared to controls [45,46]. Additionally, acupressure following laparoscopic cholecystectomy effectively alleviates acute post-operative abdominal pain compared to a sham group [47]. Similarly, acupressure at the Lanwei point (Ex-Le7) significantly lowers post-appendectomy VAS pain scores [48]. While our findings are consistent with these studies, it is important to consider variations in acupressure protocols, participant characteristics, and pain measurement timings.

### 4.1 Study implications

This study offers valuable evidence on the effectiveness of acupressure for individuals with IBD. Its observed benefits, negligible adverse effects, ease of use, affordability, and non-invasive nature make acupressure a suitable complementary alternative therapy to enhance the quality of life and reduce pain severity in IBD patients. Furthermore, this approach can be integrated into nursing school curricula and educational programs, particularly in developing countries like Iran [49,50]. Nurses and health professionals can also educate patients and families about the advantages of self-acupressure, encouraging its continued practice at home.

### 4.2 Study novelties and limitations

This study represents the first attempt to assess quality of life and pain in IBD patients undergoing an acupressure program. We employed a sham comparator to strengthen the study's internal validity, which is a notable aspect of this research. Additionally, conducting acupressure sessions at patients' homes saves time and reduces costs associated with healthcare facility visits, making it particularly beneficial for those in low-income countries or during situations like the COVID-19 lockdown when access to clinics is limited. We also monitored patients' at-home compliance through daily phone calls, addressing a significant aspect often overlooked in previous studies.While the aforementioned novelties and strengths are noteworthy, the results should be interpreted with caution due to certain limitations. Specifically, the generalizability of the findings is constrained by the small sample size of Iranian patients.Also, We could not conduct long-term follow-up, so we documented the study outcomes upon completion. However, we relied solely on IBDQ-9 and VAS to assess outcomes, which may provide a limited perspective on overall quality of life and pain severity.

### 4.3 Suggestions for future studies

To assess the sustainability of the findings, long-term studies with larger sample sizes are needed to explore the effects of acupressure programs in comparable populations. Trustworthy analyses of outcomes, particularly quality of life (QoL), would benefit from data triangulation and feedback from multiple informants, such as family members, alongside questionnaires. Future research should also employ more targeted tools for evaluating QoL in inflammatory bowel disease (IBD), such as the IBD quality-of-life questionnaire (IBDQOL-22) [51]. Additionally, it is important to clarify the effects of similar interventions on other health outcomes like depression and self-care.

## 5 Conclusion

The self-acupressure program, involving acupressure at four points three times daily for four weeks, successfully improved the QoL and reduced pain severity in individuals with IBD.

## Funding sources

This trial was financially funded by the Research and Technology Chancellor of Aja University of Medical Sciences, Tehran, Iran (No. 599579).

## Declaration of generative AI in scientific writing

None used.

## Author contributions

Study design: NR, ZA, AHP, FT, MI; Manuscript writing: NR, ZA, AHP, FT, MI; Data collection: NR, ZA; Data Analysis: NR, AHP, FT, MI; Study supervision: NR, AHP, FT, MI; Critical revisions for important intellectual content: NR, ZA, AHP, FT, MI.

## Conflict of interest

The authors declare that they have no known competing financial interests or personal relationships that could have appeared to influence the work reported in this paper.
