# Supplementary material for: Effects of self-acupressure on quality of life and abdominal pain severity among patients with inflammatory bowel disease: A randomized sham-controlled trial
Source: J Ayurveda Integr Med. 2025 Mar 25;16(2):101080. doi: 10.1016/j.jaim.2024.101080 (PMC11984590; doi:10.1016/j.jaim.2024.101080)
Supplement: Multimedia component 1 [file mmc1.docx]

**Demographic-clinical questionnaire**

Dear research assistant

Please complete the following eight items by interviewing the patients and extracting information from their medical records. Another assistant will fill out the first two items.

Completion date: ……………………….…..

| **Questionnaire code** | **…………………..** |
| --- | --- |
| **Study group** | a) Acupressure b) Sham |
| **1) Age (year)** | **……………………** |
| **2) Duration of IBD (years)** | **……………………** |
| **3) Gender** | a) Male b) Female |
| **4) Marital Status** | a) Single b) Married c) Divorced |
| **5) Job status** | a) Jobless b) Employed c) Retired |
| **6) Educational level** | a) Below diploma b) Diploma c) Academic |
| **7) History of hospitalization** | a) Yes b) No |
| **8) Diet type** | a) Regular b) Diabetic c) Low salt-fat  a) Fast food b) Without lactose/spices |
| **Abbreviation:** IBD, Inflammatory bowel disease | |

**Quality of life questionnaire for patients with inflammatory bowel disease (IBDQ-9)**

Completion date: ……………………….…..

| **Questionnaire code** | **…………………..** |
| --- | --- |
| **Study group** | a) Acupressure b) Sham |

Dear patient

The following questions concern your bowel problems and how they have affected your life over the past four weeks. Please read each question carefully and select one answer. Choose the best answer if you are unsure about a question's response.

| **No** | **Questions** | **Responses** | |
| --- | --- | --- | --- |
| **1** | **1- What was the number of times you had bowel movements in the last four weeks?** | a) More than ever | b) Very much |
|  |  | c) Much | d) Increased moderately |
|  |  | e) Increased a little | f) Not increased almost |
|  |  | g) Not increased at all |  |
| **2** | **How much have you felt sluggish and tired during the last four weeks?** | a) More than ever | b) Very much |
|  |  | c) Much | d) Moderate |
|  |  | e) Little | f) Very little |
|  |  | g) None |  |
| **3** | **How has your physical energy been during the last four weeks?** | a) Less than ever | b) Very low |
|  |  | c) Low | d) Moderate |
|  |  | e) Good | f) Very good |
|  |  | g) Excellent |  |
| **4** | **During the last four weeks, how often have you had to cancel or delay a task or social appointment due to your illness?** | a) More than ever | b) Very much |
|  |  | c) Much | d) Moderate |
|  |  | e) Little | f) Very little |
|  |  | g) None |  |
| **5** | **How much have you experienced stomach cramps during the last four weeks?** | a) More than ever | b) Very much |
|  |  | c) Much | d) Moderate |
|  |  | e) Little | f) Very little |
|  |  | g) None |  |
| **6** | **How unwell have you been during the past four weeks?** | a) More than ever | b) Very much |
|  |  | c) Much | d) Moderate |
|  |  | e) Little | f) Very little |
|  |  | g) None |  |
| **7** | **How often have you had problems with excessive gas excretion from the intestines in the last four weeks?** | a) More than ever | b) Very much |
|  |  | c) Much | d) Moderate |
|  |  | e) Little | f) Very little |
|  |  | g) None |  |
| **8** | **How much have you had problems with abdominal bloating during the last four weeks?** | a) More than ever | b) Very much |
|  |  | c) Much | d) Moderate |
|  |  | e) Little | f) Very little |
|  |  | g) None |  |
| **9** | **How satisfied and happy have you been during the last four weeks?** | a) More than ever | b) Very much |
|  |  | c) Much | d) Moderate |
|  |  | e) Little | f) Very little |
|  |  | g) None |  |

**Visual analog scale (VAS) for pain evaluation**

Completion date: ……………………….…..

| **Questionnaire code** | **…………………..** |
| --- | --- |
| **Study group** | a) Acupressure b) Sham |

Dear patient

Below is a calibrated line of ten numbers, with two end-points of zero, labeled by “No pain” to 10, marked by “Worst pain possible.” Please determine your pain level in the abdominal site, indicating a position in the line.


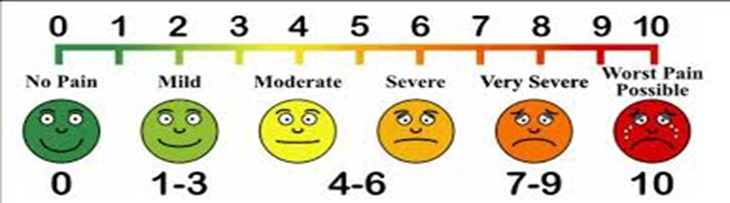


**Adverse effects form**

Completion date: ……………………….…..

| **Questionnaire code** | **…………………..** |
| --- | --- |
| **Study group** | a) Acupressure b) Sham |

Dear research assistant

Please report any potential adverse effects of interventions during the daily follow-up calls.

1. **………………………………………………..**
2. **………………………………………………..**
3. **………………………………………………..**
4. **………………………………………………..**
5. **………………………………………………..**
6. **………………………………………………..**
7. **………………………………………………..**
8. **………………………………………………..**
9. **………………………………………………..**
10. **………………………………………………..**
